# Moderate-severe postoperative pain in patients undergoing video-assisted thoracoscopic surgery: A retrospective study

**DOI:** 10.1038/s41598-020-57620-8

**Published:** 2020-01-21

**Authors:** Kai Sun, Daiyun Liu, Jie Chen, Shui Yu, Yongyu Bai, Congcong Chen, Yu Yao, Lina Yu, Min Yan

**Affiliations:** Department of Anesthesiology and Pain Medicine, Second Affiliated Hospital, Zhejiang University School of Medicine, NO. 88 Jiefang Road, Hangzhou, 310009 China

**Keywords:** Risk factors, Pain

## Abstract

Moderate-severe pain after surgical procedures is associated with decreased quality of life and increased costs. This study aimed to identify the incidence and predictive factors of moderate-severe postoperative pain within 48 hours following video-assisted thoracoscopic surgery (VATS) in a tertiary hospital. A retrospective cohort analysis was performed using medical records of adult patients who underwent VATS between January 2015 and December 2016. Logistic regression was performed to identify predictive factors for moderate-severe pain (visual analogue scale, VAS ≥ 4) within 24 hours and within 48 hours postoperatively. Of the 1164 participants, the incidence of moderate-severe pain was 12.7% within the first 24 hours and 15.6% within the first 48 hours after surgery. In multivariable analysis, the independent risk factors related to moderate-severe pain within 24 hours after surgery were younger age, increased body mass index, preoperative pain within 1 month and history of smoking. The risk factors for moderate-severe acute pain within 48 hours were almost the same, except that the number of chest tubes were also included. Moderate-severe postoperative pain following VATS is not rare, and presence of several risk factors deserves more aggressive pain management strategies perioperatively.

## Introduction

Moderate-severe pain after surgical procedures is associated with prolonged hospital stays, readmissions, patient dissatisfaction, development of chronic pain, decreased quality of life and increased costs^1,2^. It is widely accepted that better management of postoperative pain results in better patients’ recovery.

Video assisted thoracoscopic surgery (VATS), compared to conventional thoracotomy, is notable for less tissue trauma, improved postoperative respiratory function, and increased tolerability for the patients^3^. Although potentially less postoperative pain was generally expected as one of the benefits of VATS^4^, many patients still suffer moderate to severe pain postoperatively^2,5–7^. There are several reasons contributing to this unsatisfactory situation, one of them being that some patients may have certain risk factors for occurrence of more severe postoperative pain^8^. Early identification of patients at risk may help to allow early application of effective treatment strategies to prevent unexpected suffering in these patients.

Previously, several studies have focused on the predictors of postoperative pain and demographic, social psychological and clinical factors have been identified contributing to the development of severe postoperative pain^5,6,8,9^. However, most of them are cohorts with mixed surgical procedures while VATS is not included. Moreover, various subtypes of VATS (uniportal, multiportal and robotic) exist currently, and the lack of guideline results in variation of analgesia regimens and effectiveness of analgesia in different medical centers^10^. To date, the predictive factors of moderate-severe postoperative pain following VATS were not reported, which is important for individual-specific pain management perioperatively.

This study was therefore conducted to investigate the incidence and risk factors of moderate-severe postoperative pain over the first 48 hours following VATS in a tertiary hospital.

## Results

A total of 1177 patients underwent VATS during the study period. 13 patients were excluded because of bilateral surgery (n = 3), conversion to thoracotomy (n = 9) and VATS together with inguinal hernia repair (n = 1), leaving 1164 patients for analysis. The average age of the study population was 58 ± 11 years and 48.4% of study participants were male. In this population, 1 (0.1%) patient underwent pneumonectomy, 840 (72.2%) patients underwent lobectomy, 108 (9.3%) patients underwent segmentectomy, 180 (15.5%) patients underwent wedge resection and 35 (3.0%) patients underwent bullectomy. The preoperative and intraoperative characteristics of 1164 participating patients are presented in Table 1. Characteristics were compared between patients with or without moderate-severe pain within 24 hours postoperatively (Table 1).

**Table 1:** Demographic and perioperative characteristics of 1164 participating patients.

Variables		Moderate-severe pain within 24 h	No moderate-severe pain within 24 h	P
Age (y)	58 ± 11	54.9 ± 12.5	58.9 ± 11.0	<0.001
Gender (M, %)	563 (48.4%)	82 (55.4%)	481 (47.3%)	0.067
BMI (kg/m^2^)	22.8 ± 3.1	23.4 ± 3.1	22.7 ± 3.1	0.013
Educational level (n, %)				0.061
Preliminary school	444 (38.1%)	42 (28.4%)	402 (39.6%)	
Middle school	324 (27.8%)	46 (31.1%)	278 (27.4%)	
High school	178 (15.3%)	25 (16.9%)	153 (15.1%)	
College	218 (18.7%)	35 (23.6%)	183 (18.0%)	
Hypertension (n, %)	360 (30.9%)	48 (32.4%)	312 (30.7%)	0.672
Diabetic mellitus (n, %)	113 (9.7%)	14 (9.5%)	99 (9.7%)	0.913
Coronary artery disease (n, %)	97 (8.3%)	12 (8.1%)	85 (8.4%)	0.915
Smoking (n, %)	374 (32.1%)	61 (41.2%)	313 (30.8%)	0.011
Preoperative pain within 1 month (n, %)	209 (18.0%)	36 (24.3%)	173 (17.0%)	0.031
ASA				0.051
I	22 (1.9%)	6 (4.1%)	16 (1.6%)	
II	1084 (93.1%)	138 (93.2%)	946 (93.1%)	
III	58 (5.0%)	4 (2.7%)	54 (5.3%)	
Subtypes of surgery				0.035
Uni-port VATS (n, %)	431 (37.3%)	66 (45.2%)	365 (36.2%)	
Multi-port VATS (n, %)	724 (62.7%)	80 (54.8%)	644 (63.8%)	
Intraoperative fluid resuscitation (L)	1.7 ± 0.6	1.7 ± 0.5	1.7 ± 0.6	0.397
Intraoperative morphine consumption (mg•kg^−1^•h^−1^)	0.8 ± 0.2	0.8 ± 0.2	0.8 ± 0.2	0.946
Surgery duration (h)	2.7 ± 1.1	2.7 ± 1.3	2.7 ± 1.0	0.636
Anesthesia duration (h)	3.4 ± 1.5	3.4 ± 2.4	3.4 ± 1.3	0.988
Malignant tumor (n, %)	944 (18.1%)	118 (79.7%)	826 (81.3%)	0.649
Number of chest tubes (1/2/3)				0.370
1	795 (68.3%)	94 (63.5%)	701 (69.0%)	
2	368 (31.6%)	54 (36.5%)	314 (30.9%)	
3	1 (0.1%)	0	1 (0.1%)	
Chest tube in place (days)	5.8 ± 4.3	5.8 ± 4.1	5.8 ± 4.3	0.930
Postoperative morphine consumption within 24 h (mg)	45.1 ± 13.5	52.2 ± 14.2	44.1 ± 13.0	< 0.001

ASA, American Society of Anesthesiologists; VATS, video-assisted thoracoscopic surgery.

The incidence of moderate-severe pain was 12.7% within the first 24 hours and 15.6% within the first 48 hours postoperatively. The average VAS was 4 (4–5) for patients with moderate-severe pain vs. 2 (1–2) for patients without moderate-severe pain. Patients with moderate-severe pain pressed the PCA/PVB button significantly more frequently (3.3 ± 5.3 vs. 1.4 ± 2.4, P = 0.003), and required more morphine than patients without moderate-severe pain (Table 1).

In the study population, ICNB combined with PCA was applied in 1095 patients (94.1%), while PVB combined with systemic analgesia was applied in 69 patients (5.9%) (Table 2). Inadequate analgesia occurred in a comparable proportion of patients in the two analgesia regimen groups (P > 0.05, Table 2). In univariable analysis, development of moderate-severe postoperative pain within the postoperative 24 hours or 48 hours was associated with younger age, increased BMI, better education, preoperative pain within 1 month, lower ASA grade, subtypes of surgery, smoking history and number of chest tubes (Tables 3 and 4). Comorbidities and intraoperative variables were similar between patients with or without insufficient analgesia.

**Table 2:** Comparisons of postoperative pain during the postoperative 48 hours between different postoperative analgesia schedules.

	All patients (N = 1164)	PVB combined with systemic analgesia (n = 69)	ICNB combined with PCA (n = 1095)	P
Moderate-severe pain in Day 1	148 (12.7%)	12 (17.4%)	136 (12.4%)	0.229
Moderate-severe pain in Day 2	64 (5.5%)	4 (5.8%)	60 (5.5%)	0.911
Moderate-severe pain within 2 days	182 (15.6%)	14 (20.3%)	168 (15.3%)	0.272

PVB, paravertebral block; ICNB, intercostal nerve block; PCA patient-controlled analgesia.

**Table 3:** Univariable and multivariable analysis of the related factors for moderate-severe postoperative pain within 24 hours.

	Univariable analysis		Multivariable analysis	
	OR (95% CI)	P	OR (95% CI)	P
Age	0.97 (0.96–0.99)	<0.001	0.96 (0.95–0.98)	<0.001
Gender (M)	1.38 (0.98–1.96)	0.067	/	
Body mass index	1.07 (1.01–1.13)	0.013	1.10 (1.04–1.16)	0.001
Educational level			/	
Preliminary school	0.61 (0.41–0.89)	0.009	/	
Middle school	1.20 (0.82–1.74)	0.346	/	
High school	1.15 (0.72–1.82)	0.563	/	
College	1.41 (0.93–2.13)	0.102	/	
Hypertension	1.08 (0.75–1.57)	0.672	/	
Diabetic mellitus	0.97 (0.54–1.74)	0.913	/	
Coronary artery disease	0.97 (0.51–1.82)	0.915	/	
Smoking	1.58 (1.11–2.24)	0.012	1.86 (1.29–2.70)	0.001
Preoperative pain within 1 month	1.57 (1.04–2.36)	0.032	1.55 (1.01–2.39)	0.044
ASA	0.44 (0.21–0.90)	0.025	/	
Intraoperative fluid resuscitation	0.87 (0.64–1.20)	0.396	/	
Intraoperative morphine consumption	0.97 (0.46–2.08)	0.946	/	
Surgery duration	0.96 (0.81–1.14)	0.636	/	
Anesthesia duration	1.00 (0.89–1.12)	0.988	/	
Analgesia schedules			/	
ICNB combined with PCA	0.67 (0.35–1.29)	0.232	/	
PVB combined with systemic analgesia	1.49 (0.78–2.84)	0.232	/	
Subtypes of surgery			/	
Uni-port VATS (n, %)	1.46 (1.03–2.07)	0.036	/	
Multi-port VATS (n, %)	0.69 (0.48–0.98)	0.036	/	
Number of chest tubes	1.27 (0.89–1.82)	0.191	/	

ASA, American Society of Anesthesiologists; ICNB, intercostal nerve block; PCA patient-controlled analgesia; PVB, paravertebral block; OR, odds ratio; CI, confidence interval. In adjusted analysis, the following variables were included in analysis: age, gender, body mass index, educational level, smoking, preoperative pain within 1 month and ASA.

**Table 4:** Univariable and multivariable analysis of the related factors for moderate-severe postoperative pain within 48 hours.

	Univariable analysis		Multivariable analysis	
	OR (95% CI)	P	OR (95% CI)	P
Age	0.96 (0.97–0.99)	0.001	0.97 (0.96–0.99)	<0.001
Gender (M)	1.26 (0.92–1.74)	0.148	/	
Body mass index	1.05 (1.00–1.10)	0.059	1.07 (1.02–1.13)	0.009
Educational level			/	
Preliminary school	0.64 (0.46–0.90)	0.011	/	
Middle school	1.18 (0.84–1.67)	0.337	/	
High school	1.06 (0.69–1.64)	0.793	/	
College	1.43 (0.98–2.08)	0.066	/	
Hypertension	1.09 (0.77–1.52)	0.636	/	
Diabetic mellitus	0.95 (0.55–1.64)	0.855	/	
Coronary artery disease	1.45 (0.86–2.44)	0.160	/	
Smoking	1.32 (0.95–1.83)	0.101	1.53 (1.09–2.16)	0.015
Preoperative pain within 1 month	1.46 (1.00–2.15)	0.051	1.50 (1.01–2.23)	0.046
ASA	0.50 (0.26–0.97)	0.039	/	
Intraoperative fluid resuscitation	0.80 (0.60–1.08)	0.149	/	
Intraoperative morphine consumption	1.14 (0.57–2.26)	0.717	/	
Surgery duration	0.95 (0.81–1.11)	0.490	/	
Anesthesia duration	0.98 (0.87–1.10)	0.699	/	
Analgesia schedules			/	
ICNB combined with PCA	0.71 (0.39–1.31)	0.274	/	
PVB combined with systemic analgesia	1.41 (0.76–2.58)	0.274	/	
Subtypes of surgery			/	
Uni-port VATS (n, %)	1.72 (1.25–2.36)	0.001	/	
Multi-port VATS (n, %)	0.58 (0.42–0.80)	0.001	/	
Number of chest tubes	1.42 (1.02–1.96)	0.037	1.48 (1.06–2.07)	0.021

ASA, American Society of Anesthesiologists; ICNB, intercostal nerve block; PCA patient-controlled analgesia; PVB, paravertebral block; CI, confidence interval. In adjusted analysis, the following variables were included in analysis: age, gender, body mass index, educational level, smoking, preoperative pain within 1 month, ASA, intraoperative fluid resuscitation and number of chest tubes.

**Figure 1:**
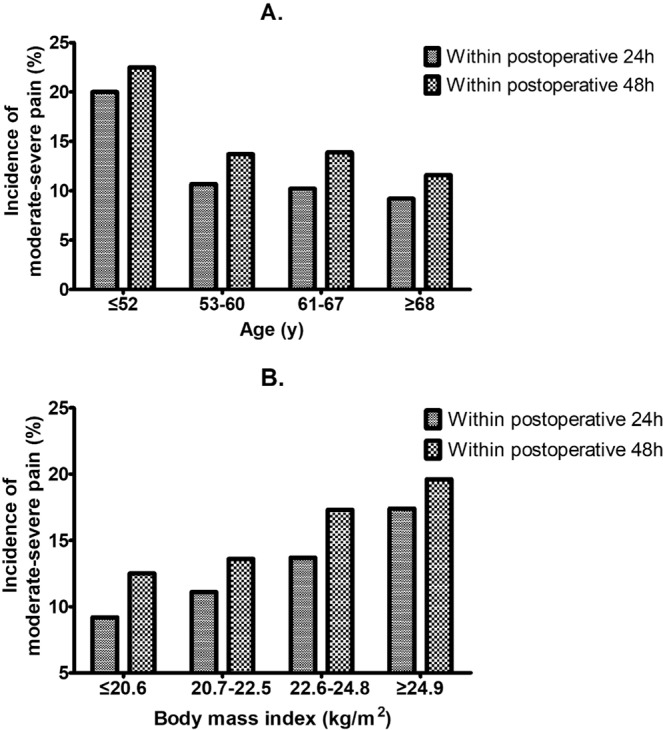
The incidence of moderate-severe acute pain after VATS in four different age groups (quartiles, A) and different body mass index (BMI) groups (quartiles, B). Age of the four quartile groups were ≤52, 53–60, 61–67 and ≥68 and pain significantly increased at age ≤52 years. BMI of the four quartile groups were ≤20.6, 20.7–22.5, 22.6–24.8 and ≥24.9 and pain significantly increased as BMI grew.

In multivariable analysis, the independent risk factors related to moderate-severe pain within 24 hours after surgery were younger age (OR, 0.96; 95% CI, 0.95 to 0.98, P < 0.001), increased BMI (OR, 1.10; 95% CI, 1.04 to 1.16, P = 0.001), preoperative existing pain within 1 month (OR, 1.55; 95% CI, 1.01 to 2.39, P = 0.044) and smoking history (OR, 1.86; 95% CI, 1.29 to 2.70, P = 0.001). As shown in Table 4, the risk factors for moderate-severe acute pain within 48 hours were almost the same, except that the number of chest tubes (OR, 1.48; 95% CI, 1.06 to 2.10, P = 0.019) were also included (Table 4, Fig. 1).

In regards to early postoperative outcomes, occurrence of moderate-severe pain was not associated with increased incidence of nausea or vomiting. And postoperative hospital days were comparable between patients with or without adequate analgesia. However, moderate-severe acute pain lead to significantly lower patient satisfaction (50.5% vs. 90.6%, P < 0.001) (Table 5).

**Table 5:** Relationship between moderate-severe pain after VATS and postoperative outcomes.

	Moderate-severe acute pain within 24 h		P	Moderate-severe acute pain within 48 h		P
	Yes	No		Yes	No	
Postoperative nausea	36 (24.3%)	222 (21.9%)	0.498	56 (30.8%)	274 (27.9%)	0.431
Postoperative vomiting	22 (14.9%)	159 (15.6%)	0.806	35 (19.2%)	189 (19.2%)	0.996
Postoperative days	8.3 ± 4.4	8.8 ± 6.7	0.360	8.1 ± 4.0	8.9 ± 6.8	0.134
Satisfaction	/	/	/	92 (50.5%)	887 (90.6%)	<0.001

## Discussion

This large retrospective cohort study revealed that 15.6% of patients suffered from moderate-severe pain over 48 hours after VATS. Our findings indicate that patients with younger age, increased BMI, preoperative existing pain within 1 month, history of cigarette smoking, and increased number of chest tube placement had a statistically significant increased risk for pain following VATS. The presence of these risk factors should require more aggressive pain management strategies perioperatively.

Acute pain following VATS is not rare because of multiple muscle incisions and irritation of the pleura with chest tubes. In this retrospective study of 1164 adult patients undergoing VATS, the prevalence of moderate-severe postoperative pain over 48 hours was 15.7%. This finding differs from the results obtained by Emine *et al*.^6^, who reported a higher incidence of pain (59%). This is mainly owing to different patient characteristics, diverse types of surgery performed and distinct follow-up periods.

Elevated BMI as well as preoperative existing pain have previously been reported to be associated with increased postoperative pain in different types of surgeries other than VATS^8,13–16^. Our study was the first to demonstrate that these associations also present in patients undergoing VATS and that patients with high BMI and pre-existing pain deserved additional care. A 1 kg/m^2^ increase in BMI was associated with an increased risk of postoperative pain by 10% (OR, 1.10; 95% CI, 1.04 to 1.16), and the presence of preoperative pain increased the risk of pain by 55% (OR, 1.55; 95% CI, 1.01 to 2.39, P = 0.044). A possible explanation for the relationship of BMI to moderate-severe acute pain may be that high BMI is associated with development of postoperative complications, such as wound infection, contributing to frequent debridement, change of dressing and increased postoperative pain^17^. Besides, opioids such as sulfentanil as we used in this study were distributed at least as extensively in the excess body mass as in the lean tissues, therefore the same dose of opioids might be less effective in obese patients^18,19^. Additionally, obesity and preoperative pain were both characterized by elevated proinflammatory cytokines and subclinical systemic inflammation, leading to decreased pain threshold^15,20–22^. Further study is needed to elucidate the potential mechanisms behind our observations.

Younger age is another consistent risk factor for moderate-severe pain following VATS, similar to a study including robotic-assisted thoracic surgery, VATS and open lobectomy^5^, but in contrast to a recent study reporting no age-related differences by limited sample size^6^. This finding could be explained by the fact that the old had higher pain threshold, more peripheral neuropathy and less scar with less stiffness, contributing to less pain. Besides, elderly patients showed increased sensitivity to perioperative opioids with pharmacokinetic and psychosocial mechanisms^23,24^. More smokers underwent VATS compared with non-smokers, and it is therefore important to detect whether the increased risk for postoperative pain was smoking-related actually or if there was other explanations. And in VATS, the association between smoking history and postoperative pain was controversial^25,26^. The multiple analysis of this study indicated that presence of previous history of smoking increased the risk of moderate-severe acute pain following VATS by 86% (OR, 1.86; 95% CI, 1.29 to 2.70, P = 0.001). The potential mechanism was that chronic nicotine exposure was associated with pain intolerance and mechanical hyperalgesia by elevating spinal dynorphin and cytokines in rats^27^. Chest tube placement contributes to postoperative pain, and early removal of chest tubes was recommended^28^.

Even though female sex was shown to be related to increased pain and narcotic use in various procedures, this was not seen in our cohort. Diabetes and educational level also did not have any association with pain either. Despite our expectations, there was no association between the analgesic methods and moderate-severe acute pain, which might be confounded by the severe imbalance of group distribution and variability in analgesia modalities of different anesthesiologists. Further studies comparing the efficiency and safety of different analgesia strategies in VATS should be undertaken in the future.

We did not find a significant association between moderate-severe pain and postoperative hospital days. This could be explained, at least in part, by the prolonged hospital days (8–9 days) in our institution. In most western studies, the hospital days after VATS are much shorter (1–4 days), and the pain level at days 1–2 may have had more influence on hospital stay if the average length of stay were shorter. Our data showed that occurrence of moderate-severe acute pain following VATS was negatively related to patients’ satisfaction rate (90.6% vs. 50.5%, P < 0.001). So investigation of risk factors of postoperative pain is appealing. The independent factors in this study were simple and were confirmed in two models (pain within postoperative 24 hours and postoperative 48 hours) to be well correlated with risks of acute pain. Since patients with younger age, increased BMI, preoperative existing pain within 1 month, history of cigarette smoking, and increased number of chest tube placement have an extremely high risk, they deserve extra attention to perioperative pain management. Preoperatively, patient expectations should be set according to the anticipated pain intensity and postoperative analgesia protocol. Epidural analgesia and auxiliary analgesia methods should be considered, and sometimes consultation with a pain physician is necessary.

Several limitations are worth mentioning. First is the retrospective nature of this study. It is possible that confounding factors for pain could not obtained in our study, such as psychological variables. Some additional complexities to each case including different surgeons/anesthesiologists, distinctive incisions and variance of postoperative courses contributing differently to pain were not taken into account. And the anesthetics in this trial were not standardized, but followed routine practice. For example, at the end of surgery, three different kinds of analgesics were administered for different patients. Prospective studies controlling for these variables were recommended. Secondly, VAS is subjective and only the maximum score within 24 hours and 48 hours was recorded and analyzed, while the median of pain score was inaccessible during the study period. However, the VAS is used widely across institutions for pain assessment, and the patients were followed up by the same nurse during the study period. Besides, patient demographics and management varies from centers to centers, so our results should be drawn with caution.

In conclusion, moderate-severe acute pain occurred in 15.6% of adult patients undergoing VATS and was associated with patients’ dissatisfaction. Younger age, increased BMI, preoperative existing pain within 1 month, history of cigarette smoking, and increased number of chest tube placement were risk factors of postoperative pain. Gender and educational level did not appear to be associated with acute pain in patients undergoing VATS.

## Methods

This single-center retrospective study was approved by institutional review board (IRB) of the Second Affiliated Hospital of Zhejiang University, School of Medicine. All methods were performed in accordance with the guidelines and regulations. The requirement for informed consent was waived by the IRB based on the retrospective nature of the study and the minimum intrusion on participants’ privacy. All adult patients undergoing elective VATS for lung disease between January 2015 and December 2016 in our institution were identified. Excluded criteria was bilateral surgery, conversion to thoracotomy and another procedure performed at the same time.

In VATS procedures, rib retractors were not applied. Single-port VATS and multi-port VATS were selectively used decided by the attending surgeon according to patient’s condition and personal preference. The maximum size of the utility incision was 1–4 centimeters routinely in our institution. Disposable drainage tubes with a diameter of 7.33 mm (F22, Mclean Medical Device Products Co., Ltd, Suzhou) were used at the end of surgery.

Anesthetic management followed the standard care at our institution. Anesthesia was induced and maintained with intravenous and inhalational anesthetics combined with sulfentanyl/remifentanil/fentanyl. Muscle relaxants were administered as indicated. Intraoperative opioids were converted to morphine so the dosages could be compared^11^. In addition to standard monitors, central venous catheter and an arterial catheter were placed. Bispectral index was monitored routinely. Postoperatively, patients were transferred to the postanesthesia care unit or intensive care unit as needed where they were continuously monitored. Postoperative care was standardized as per institutional routines.

Patients received either of the two analgesia regimens, continuous paravertebral block (PVB) by the surgeon under video-assisted vision combined with a single bolus of intravenous analgesic (such as pentazocine, dynastat or dezocine), or a single-shot intercostal nerve block (ICNB) by the surgeon combined with patient-controlled analgesia (PCA) with opioids. Continuous PVB protocol was 0.15–0.20% ropivacaine with a continuous dose of 5 mL and a bolus dose of 1 mL with a lock-out of 15 minutes. A single bolus of 15–20 mL ropivacaine were administered to make sure of the appropriate location of the catheter after placement at the end of surgery. ICNB was performed using 0.75% ropivacaine 10 mL under video-assisted vision. The PCA protocol was 0.1 mg of sulfentanyl diluted to 100 mL with a continuous dose of 0.03–0.05 mL•kg^−1^•h^−1^ and a bolus dose of 0.02–0.03 mL•kg^−1^•h^−1^ with a lock-out of 15 minutes. PCA/PVB pump was attached to the patient immediately after surgery and was stopped after 48 hours. If the analgesia was inadequate (visual analog scale, VAS ≥ 4) during the postoperative period, patients were recommended to press the PCA/PVB button. And if relief was not obtained, additional analgesics were given intravenously as rescue. All patients received tropisetron at the end of surgery to prevent postoperative nausea and vomiting, additional bolus was supplemented as needed.

Patients’ demographic characteristics (age, gender, body mass index [BMI] and educational level), comorbidity (hypertension, diabetes mellitus and coronary artery disease), cigarette smoking history and American Society of Anesthesiologists (ASA) grade were retrospectively collected. If the patient has been smoking cigarette everyday for more than 1 year, no matter he quit smoking or not perioperatively, he was considered as having smoking history in this study. And preoperative pain experience within 1 month before surgery was collected from medical records. It was evaluated and recorded by the nurses in wards on the first day of admission in our institution, defined as continuous pain feeling lasting for more than 1 day during the past 1 month, no matter of the degree, location and character of the pain. Intraoperative characteristics included subtypes of surgery (single-port VATS or multi-port VATS), volume of fluid resuscitation, morphine consumption, surgery duration, anesthesia duration, pathological diagnosis and total number of chest tube placement. The postoperative days of chest tube in place were also recorded. In our institution, postoperative pain and related variables were followed up every day by a nurse at 24 and 48 hours after surgery. The most severe pain at rest since the last visit evaluated by VAS (0 = no pain, 10 = worst pain) and total dose of morphine requirement were recorded. The primary outcome was the incidence of moderate-severe pain (VAS ≥ 4) within 24 hours and within 48 hours postoperatively. Secondary outcomes included incidence of postoperative nausea and vomiting, postoperative hospital days and patient satisfaction. Patient satisfaction rate was defined by the incidence of the “extremely satisfactory” on a 5-point scale (1 = unsatisfactory, 5 = extremely satisfactory) as previously described^12^.

Data were reported as mean value ± standard deviation or median (interquartile range) for continuous variables, or percentages for categorical variables. Student t or Mann Whitney U test were performed for continuous variables and the Chi square test or Fisher’s test was performed for categorical variables as appropriate. Independent risk factors were identified using a multivariable logistic regression model that include preoperative and intraoperative variables with P < 0.15 in univariable analysis and odds ratio (OR) and 95% confidence interval (CI) were calculated. All tests were performed using SPSS, version 22.0 (IBM, Armonk, NY), with P < 0.05 considered to be statistically significant.

## Data Availability

The datasets used and/or analyzed during the current study are available from the corresponding author upon reasonable request.
